# Transactions of the American Dental Society

**Published:** 1863-11

**Authors:** 


### TRANSACTIONS OF THE AMERICAN DENTAL SOCIETY.

The Transactions of the last meeting of this body are now ready for distribution to all the members of the Association who have paid their annual dues, or may now pay the same; and also for sale to any who may desire to purchase them. Every member of any Association represented should have a copy of these Transactions. The Publication Committee have, in accordance with the suggestions made, had quite a number bound up with all the former Transactions, for those who may prefer to have them all in one volume rather than two. Those having the former volume, of course will not wish this double volume; for all such the present Transactions are bound separate. We shall without delay send them to all, entitled to them, and any others desiring to obtain them can do so through any of the Publication Committee.

The price is for the single volume $2 00, and for the double volume or the whole from the beginning, $3 00.

The Publication Committee are:—J. Taft, H. A. Smith, H. R. Smith, of Cincinnati; C. W. Spalding, of St. Louis, Mo.; Wm. A. Pease, of Dayton, O. T.

